# Protocol for multi-modal single-cell RNA sequencing on *M. tuberculosis*-infected mouse lungs

**DOI:** 10.1016/j.xpro.2023.102102

**Published:** 2023-02-07

**Authors:** Davide Pisu, David G. Russell

**Affiliations:** 1 Microbiology and Immunology, College of Veterinary Medicine, Cornell University, Ithaca, NY 14853, USA

**Keywords:** Bioinformatics, Cell Biology, Flow Cytometry/Mass Cytometry, Genomics, Immunology, Microbiology, Sequencing, Single Cell

## Abstract

To elucidate how different immune cells contribute to control or progression of *M. tuberculosis* (Mtb) infection, we developed a technique to perform multi-modal single-cell RNA sequencing (scRNA-seq) from *in vivo* Mtb-infected lung macrophages. This protocol simultaneously acquires the transcriptome, surface marker expression, and bacterial phenotype of each infected cell. We describe steps for sorting Mtb-infected cells and staining with CITE-seq antibodies, as well as for methanol fixation and generation of scRNA-seq libraries. This protocol can be used on tissues derived from murine, nonhuman primate, and human infections.

For complete details on the use and execution of this protocol, please refer to Pisu et al. (2021).^1^

## Highlights

• Protocol for generation of multi-modal scRNA-seq libraries from Mtb-infected samples
• Sorting of Mtb-infected host cells based on the fitness of intracellular bacteria
• Allows the identification of permissive and controller macrophage populations
• Explore the heterogeneity of host immune responses following infection

## Abstract (editor highlights)

Publisher’s note: Undertaking any experimental protocol requires adherence to local institutional guidelines for laboratory safety and ethics.

## Graphical abstract

**Figure:**
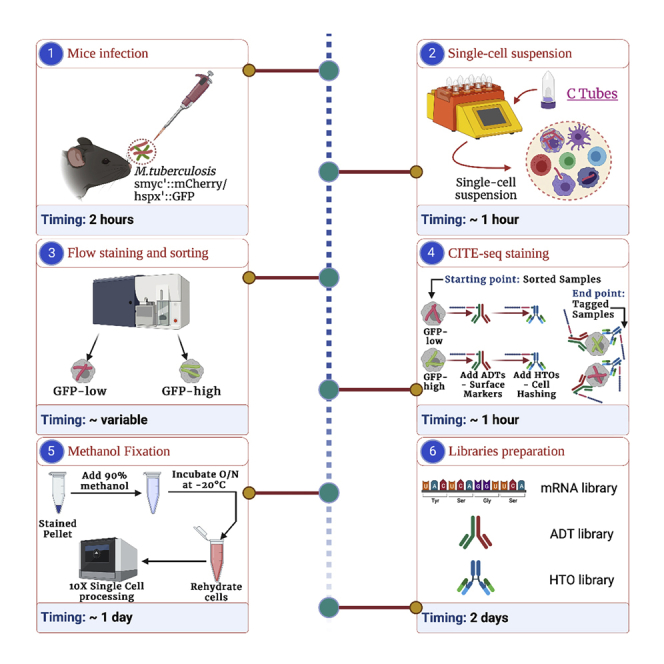

## Before you begin

Here we present a protocol to perform multi-modal single cell RNA-sequencing (scRNA-seq) on Mtb-infected mouse lungs. Because of the infectious nature of the pathogen, all steps up to methanol fixation are performed inside a BSL-3 facility. The main steps of the protocol involve mice infection, generation of a single-cell suspension from lung tissue, isolation of infected and bystander cells by fluorescence activated cell sorting, antibody staining, methanol fixation, generation of CITE-seq libraries and sequencing. Make sure all reagents required to perform the different steps are ready for use.

### Institutional permissions

Before starting the protocol, users should identify the committee approving the experiments, obtain approval for the proposed animal or human studies and confirm that all experiments conform to the relevant regulatory standards.

**CRITICAL:** For mice infection it is important to consider background, age and sex of the mice such as that they are matched as closely as possible. We typically pool 5 mice to generate an infected CITE-seq sample and 3 mice to generate a bystander CITE-seq sample to sort sufficient cells for the downstream antibody staining and library preparation steps and to minimize biological variability between infected mice.

All mice in this protocol are kept in a specific pathogen-free animal biosafety level 3 facility, in accordance with the guidelines of the Association for Assessment and Accreditation of Laboratory Animal Care.

**CRITICAL:** unless otherwise specified, all reagents and materials used in the “flow sorting, ADT-HTO staining and methanol fixation” section and below are certified DNAse- and RNAse-free.

**CRITICAL:** keep samples at 4°C or on ice whenever possible (outside of the steps involving enzymatic digestion of the tissue) to avoid unwanted alterations in the transcriptional profile of the macrophage populations.

**CRITICAL:** unless otherwise specified, all the pipette tips used in this protocol are low-retention and filtered.

## Key resources table

**Table:** 

REAGENT or RESOURCE	SOURCE	IDENTIFIER
**Antibodies**		

CD64 - PerCP/Cyanine5.5 1:200 dilution	BioLegend	Cat# 139307, RRID: AB_2561962
Mertk – PE 1:200 dilution	BioLegend	Cat# 151505, RRID: AB_2617036
SiglecF – BB515 1:200 dilution	BD Bioscience	Cat# 564514, RRID: AB_2738833
CD11c – FITC 1:200 dilution	BioLegend	Cat# 117305 RRID: AB_313774
CD45 – FITC 1:200 dilution	BD Bioscience	Cat# 561874 RRID: AB_10894189

SiglecF	BioLegend	Cat# 155513 RRID: AB_2832540
CD64	BioLegend	Cat# 139325 RRID: AB_2750367
Ly6G	BioLegend	Cat# 127655 RRID: AB_2749962
CD11c	BioLegend	Cat# 117355 RRID: AB_2750352
CD14	BioLegend	Cat# 123333 RRID: AB_2800591
CCR5	BioLegend	Cat# 107019 RRID: AB_2783049
Ly6G-Ly6C	BioLegend	Cat# 108459 RRID: AB_2783050
CD63	BioLegend	Cat# 143915 RRID: AB_2783109
F4/80	BioLegend	Cat# 123153 RRID: AB_2749986
CD38	BioLegend	Cat# 102733 RRID: AB_2750556
TLR4	BioLegend	Cat# 117614 RRID: AB_281035
CD11b	BioLegend	Cat# 101265 RRID: AB_2734152
CD16/32	BioLegend	Cat# 101343 RRID: AB_2750532
CD86	BioLegend	Cat# 105047 RRID: AB_2750348
CD1d	BioLegend	Cat# 123529 RRID: AB_2800593
CD3	BioLegend	Cat# 100251 RRID: AB_2750533
CD4	BioLegend	Cat# 100569 RRID: AB_2749956
CD8a	BioLegend	Cat# 100773 RRID: AB_2734151
HTO 1 - murine	BioLegend	Cat# 155801 RRID: AB_2750032
HTO 2 - murine	BioLegend	Cat# 155803 RRID: AB_2750033
HTO 1 - human	BioLegend	Cat# 394601 RRID: AB_2750015
HTO 2 - human	BioLegend	Cat# 394603 RRID: AB_2750016
TruStain FcX PLUS	BioLegend	Cat# 156603

**Critical commercial assays**		

Chromium Next Gem Single Cell 3′ GEM, Library & Gel Bead Kit v.3.1, 16rnx	10X Genomics	Cat# PN-1000121
Chromium Next GEM Chip G Single Cell Kit, 16 rxns	10X Genomics	Cat# PN-1000127
Single Index Kit T Set A, 96 rnx	10X Genomics	Cat# PN-1000213
Buffer EB	Qiagen	Cat# 19086
Qubit 4.0 Fluorometer	Thermo Fisher Scientific	Cat# Q33226
Qubit dsDNA HS assay kit	Thermo Fisher Scientific	Cat# Q32854
SPRIselect beads	Beckman Coulter	Cat# B23317
Protector RNAse Inhibitor	Roche	Cat# 3335402001
UltraPure Bovine Serum Albumin (BSA, 50 mg/mL)	Thermo Fisher Scientific	Cat# AM2616
DPBS – no calcium, no magnesium	Thermo Fisher Scientific	Cat# 14190144
Methanol, for HPLC	Sigma-Aldrich	Cat# 34860-100ML
Cell staining buffer	BioLegend	Cat# 420201

**Experimental models: Organisms/strains**		

C57BL/6J mice - Female – 6 Weeks old	The Jackson Laboratory	Cat# JAX:000664, RRID:IMSR_JAX:000664
*M.tuberculosis* Erdman mCherry	(Carroll et al.)^2^	N/A
*M.tuberculosis* Erdman Hspx	(Sukumar et al.)^3^	N/A
*M.tuberculosis* Erdman GFP	(Pisu et al.)^1^	N/A
*M.tuberculosis* Erdman WT	WT Erdman Tuberculosis	Cat# ATCC 35801

**Other**		

BD Tuberculin Syringe 25G	BD	Cat# E96242800JD
Isoflurane	Patterson	Cat# 14043070406
GentleMacs C tubes	Miltenyi Biotech	Cat# 130-093-237
70μM GentleMacs Strainers	Miltenyi Biotech	Cat# 130-098-462
30μM GentleMacs Strainers	Miltenyi Biotech	Cat# 130-098-458
Collagenase IV	Worthington	Cat# LS004186
Cell strainers	VWR	Cat# 10199-656 Cat# 10199-655
ACK lysis buffer	Lonza	Cat# 10-548E
EDTA	Invitrogen	Cat# AM9260G
Hepes	Gibco	Cat# 15630080
1.5 mL Eppendorf Low-bind tubes	VWR	Cat# 80077-230
2 mL Eppendorf Screw cap tubes	Thermo Scientific	Cat# 21-403-200
Nuclease Free Water	Ambion	Cat# AM9938
Low TE Buffer	Thermo Fisher	Cat# 12090-015
OADC Enrichment	VWR	Cat# 90000-614
7H9 Broth Base	Sigma	Cat# M0178-500G
7H10 Agar	VWR	Cat# 90003-728
Glycerol	VWR	Cat# 97062-454
Tyloxapol	Fisher Scientific	Cat# AC422370050
Cycloheximide	VWR	Cat# 97064-728
PBS	Corning	Cat# 21-040-CV
RNAse Zap	Fisher Scientific	Cat# AM9780

## Materials and equipment

***Alternatives:*** this protocol describes the generation of a single-cell lung suspension using a GentleMACS (with heaters) instrument from Miltenyi Biotec. If a GentleMACS is not available, it is possible to generate a single-cell suspension manually as described in previous protocols.^4^^,^^5^

***Alternatives:*** this protocol was originally performed using the 10× **Single Index** Next GEM 3′ reagent kit v 3.1 (protocol CG000206 Rev D.), which will be discontinued starting December 2022. A protocol that makes use of HTO and ADT primers compatible with dual indexing is available from Biolegend.

***Alternatives:*** this protocol requires the use of a Qubit instrument to measure the concentration (in ng/μL) of the HTO and ADT libraries. If a Qubit is not available, it is possible to submit the libraries for analysis with a Fragment Analyzer before sequencing to check both the quantity and the quality of the resulting DNA.

***Alternatives:*** in this protocol we perform HTO and ADT staining before methanol fixation. However, we successfully tested the staining of methanol-fixed samples with both HTO and ADT antibodies, therefore enabling multi-modal scRNA-seq from clinical samples, fixed at the time of collection. See later part of the protocol for detailed step-by-step instructions.

***Alternatives:*** here, we make use of TotalSeq-A antibodies from Biolegend (which contain a poly(A) tail and are compatible with any sequencing platform that relies on poly(dT) for transcript capture). However, we successfully used TotalSeq-B antibodies whose capture sequence is specific for the 10× single cell 3′ kits (v3 and v3.1).

**Table:** 7H9 Media

Reagent	Final concentration (mM or μM)	Amount
7H9 powder	N/A	2.35 gr
Glycerol	0.2% (v/v)	1 mL
Tyloxapol 20%	0.05% (v/v)	1.25 mL
OADC enrichment	10% (v/v)	50 mL
MilliQ Water	N/A	447.75 mL
**Total**	**N/A**	**500 mL**

We filter-sterilize the 7H9 media and store it at 20°C–25°C until use. While 7H9 media can theoretically be conserved indefinitely, we do not use media older than a year.

**Table:** 40% Glycerol solution

Reagent	Final concentration (mM or μM)	Amount
Glycerol	40% (v/v)	20 mL
MilliQ Water	N/A	30 mL
**Total**	**N/A**	**50 mL**

We filter-sterilize the 40% Glycerol solution and store it at 20°C–25°C until use, with storage time of up to a year.

**Table:** 7H10 Agar

Reagent	Final concentration (mM or μM)	Amount
7H10 powder	N/A	9.5 gr
Glycerol	0.5% (v/v)	2.5 mL
Cycloheximide (10 mg/mL)	100 μg/mL	5 mL
OADC enrichment	10% (v/v)	50 mL
MilliQ Water	N/A	442.5 mL
**Total**	**N/A**	**500 mL**

We first autoclave a solution containing 7H10 powder, Glycerol and MilliQ water for 15’.

After cooling down, we add OADC enrichment and Cycloheximide in a sterile hood. We then pour the 7H10 agar directly into Petri dishes, that are then stored at 20°C–25°C until use. We don’t store 7H10 plates long-term. We typically use the prepared 7H10 plates in 7–10 days.

**Table:** Infection Buffer

Reagent	Final concentration (mM or μM)	Amount
PBS 1×	N/A	49.975 mL
Tween 80	0.05% (v/v)	25 μL
**Total**	**N/A**	**50 mL**

We filter-sterilize the infection buffer and store it at 20°C–25°C until use. Maximum storage time: 6 months.

**Table:** Washing solution

Reagent	Final concentration (mM or μM)	Amount
PBS 1×	N/A	95 mL
FBS	5% (v/v)	5 mL
**Total**	**N/A**	**100 mL**

We filter-sterilize the washing solution and store it at 4°C. We don’t store the washing solution long-term. We typically use the prepared washing solution in 7–10 days.

**Table:** Dissociation Solution

Reagent	Final concentration (mM or μM)	Amount
Washing Solution	N/A	3.98 mL
Collagenase IV	250 U/mL	20 μL
**Total**	**N/A**	**4 mL**

We make stocks of Collagenase IV at 50,000 U/mL and store it at −20°C until use. The maximum recommended storage time is 1 year. Right before collecting mouse lungs, we prepare the Dissociation Solution adding Washing Solution + Collagenase IV directly into the GentleMacs C tubes under sterile conditions.

**Table:** Sorting Buffer

Reagent	Final concentration (mM or μM)	Amount
PBS 1×	N/A	47.75 mL
FBS	1% (v/v)	0.5 mL
EDTA	5 mM	0.5 mL
Hepes	25 mM	1.25 mL
**Total**	**N/A**	**50 mL**

We prepare the Sorting Buffer the day before sorting. We filter-sterilize the sorting buffer and store it at 4°C until use. We don’t store the sorting buffer long-term. We always prepare fresh sorting buffer.

**Table:** Rehydration Buffer

Reagent	Final concentration (mM or μM)	Amount
RNAse inhibitor	0.5 U/μL	25 μL
BSA	1% (v/v)	20 μL
DPBS	N/A	1,955 μL
**Total**	**N/A**	**2 mL**

We prepare the Rehydration Buffer fresh the day of the experiment and keep it on ice until needed.

## Step-by-step method details

### Prepare bacterial stocks for mice infection

**Timing: ∼ 28 days**

In this step we prepare, freeze and titer the bacterial stocks needed for mice infection.

1. Grow an aliquot of each bacterial strain needed for the experiment in a 37°C incubator, using 10 mL of 7H9 media in a 25 cm^2^ polystyrene tissue culture flask with vented cap, up to log phase (OD_600_ = 0.6–0.8).

**CRITICAL:** make sure to add the appropriate antibiotic selection marker for your strain. For our experiment we grew the hspx’::GFP/smyc’::mCherry strain in 7H9 media + 50 mg/mL of Hygromycin B. Failure to add the selection marker will result in loss of plasmid and relative fluorescence during mouse infection, and consequently it won’t be possible to discriminate between infected vs bystander cells during cell sorting.

2. After ∼ 7 days of growth (depending on the initial inoculum), check OD_600_ with a spectrophotometer. If bacteria have reached log phase go to step 3, otherwise keep growing them until they reach OD_600_ = 0.6–0.8.
3. Once bacteria have reached log phase, spin down the culture at 2,800 × *g* for 10′ at 20°C–25°C, using a 15 mL falcon tube.
4. While the bacterial cultures are spinning down, prepare and mark the amount of freezing vials that are needed to stock and freeze the bacterial samples (20 vials for each 10 mL culture).
5. Resuspend the bacterial pellet in 1 mL of 7H9 media plus antibiotic (if appropriate).
6. Using a 1 mL tuberculin syringe with a 25 gauge needle insert the syringe into the falcon tube and pass the bacterial culture in and out of the syringe for 20 times.

**CRITICAL:** proceed to the next step immediately, so that bacteria won’t have time to clump.

7. Add 9 mL of 7H9 media plus antibiotic (if appropriate) and slowly pipette the bacterial culture up and down 10 times.
8. Immediately transfer 500 μL of culture to each freezing vial.
9. Add 500 μL of a 40% glycerol stock solution to each freezing vial containing the bacterial culture (final volume = 1 mL) and pipette up and down until the bacterial stock solution appears homogenous.
10. Store the bacterial stock solutions at −80°C for 7 days.
11. After 7 days, thaw 3 randomly selected frozen vials at 20°C–25°C.

***Note:*** we wait 7 days at −80°C because after freezing some bacteria will die and we want to precisely determine the titer of the bacterial stocks prior to mice infection.

12. Transfer each bacterial stock to a 15 mL tube and passage it through a BD tuberculin syringe (25G needle) 15 times to breakup bacterial clumps.
13. Transfer 200 μL of each bacterial stock to a 96-well plate to perform serial dilutions and then plate for colony forming units (CFU) on 7H10 agar plates.
14. After 21 days, count the number of bacterial colonies to determine the titer of the frozen bacterial stocks.

### Mouse infection

**Timing: ∼ 2 h**

In this step of the protocol we are going to infect mice intranasally with 1.5 × 10^3^ CFU of our bacterial stocks.

15. Thaw the bacterial aliquots that are needed for mice infection at 20°C–25°C.

***Note:*** for cell sorting of the infected and bystander populations we infect some extra mice to use as gating and compensation controls. In the case of,^1^ we used a mouse infected with hsp60’::GFP Erdman (bacteria that constitutively express GFP), a mouse infected with smyc’::mCherry Erdman (bacteria that constitutively express mCherry) and a mouse infected with WT Erdman Mtb to be used as a compensation control for CD45 staining (See Figures S1A–S1C of^1^).

16. Transfer the bacterial cultures into 15 mL falcon tubes and passage them through a 1 mL BD Tuberculin Syringe with a 25G needle 15 times to breakup bacterial clumps.
17. Depending on the titer of the bacterial stocks calculated on step 14, calculate the number of dilutions to reach a final concentration of 5 × 10^4^ bacteria/mL needed for mice infection. Perform the dilutions in infection buffer, using 2 mL Eppendorf screw cap tubes.

**CRITICAL:** slowly pipette up and down 5–6 times when you transfer Mtb in the subsequent dilution tube to allow homogenous resuspension of the bacteria in the tube and avoid carrying over potential clumps.

18. Anesthetize the mice using an isoflurane and oxygen mixture (5% isoflurane in oxygen at 4.5 L/min; VIP 3000 isoflurane vaporizer) for ∼ 2 min, until the animals are sedated and the breath slows down.
19. 30 μL (1.5 × 10^3^ bacteria) are then administered intranasally to each mouse.

***Note:*** mice infected with different strains are kept in different cages for the duration of the infection, to prevent strain cross-contamination. We suggest preparing and labeling the cages before performing infection to avoid any potential mix-up when working with high numbers of animals.

### Generation of a single-cell suspension and antibody staining for flow sorting

**Timing: ∼ 1 h**

20. Follow steps 4–18 of the previously published protocol to obtain a lung single-cell suspension.^4^
21. Slowly remove the supernatant and resuspend in 2 mL of washing solution.
22. Transfer the lung single-cell suspensions to 2 mL Eppendorf screw cap tubes.
23. Spin down at 500 × *g* for 3′ at 20°C–25°C.

***Optional:*** resuspend in 1 mL of Fc blocking solution (Washing solution + Fc block (anti CD16/32 for mouse)) and incubate for 15′ at 4°C. Spin down at 500 × *g* for 3′ at 20°C–25°C.

***Note:*** this step is only necessary if sorting using surface markers staining to discriminate and gate on specific immune populations. *For our single-cell RNA-seq paper, we sorted directly on bacterial fluorescence to discriminate infected cells and we only used CD45 to sort the Bystander population, therefore we didn’t perform blocking.*

24. Remove the supernatant. Resuspend the lung pellet with the antibody mix in washing solution at the appropriate concentration.
25. Incubate for 30′ in the dark at 4°C.
26. Add 600 μL of washing solution and pipette mix 5–6 times.
27. Spin down at 500 × *g* for 3′ at 20°C–25°C.
28. Wash with 1 mL of washing solution and spin down again at 500 × *g* for 3’.
29. Resuspend in sorting buffer at an appropriate concentration based on the characteristics of your cell sorter (we typically resuspend the lung pellet in sorting buffer such that the sorting efficiency > 80%).
30. Pass the lung suspension through a 30 μM cell strainer to avoid the formation of clumps that can clog the nozzle of the instrument.

**CRITICAL:** keep the samples at 4°C or in ice while you setup the cell sorter.

### Flow sorting, ADT-HTO staining, and methanol fixation

**Timing: variable, depending on the number of samples and speed of the instrument**

31. Fill 1.5 mL Eppendorf low-bind tubes (one for each sorted sample) with 600 μL of Cell Staining Buffer (Biolegend).

***Note:*** this amount depends on your machine. We typically sort ∼60,000 cells in 300 μL of sorting volume.

32. Prepare the sorting gates, acquire and start sorting the samples according to the Figures S1A–S1C of the original manuscript.^1^

**CRITICAL:** Keep the sorted samples at 4°C during the sorting process if your sorter allows chilling of the output tubes.

33. Sort the cells directly into the 1.5 mL Eppendorf low-bind tubes containing 600 μL of Cell Staining Buffer (Biolegend).
34. Immediately at the end of the sorting, spin down the cells at 500 × *g* for 5′ at 20°C–25°C.
35. While the cells are spinning down, prepare the blocking solution according to manufacturer instructions. For example, if working with mice and if you have 4 sorted samples, prepare a master mix blocking solution mixing 2 μL of TruStain FcX™ PLUS (anti-mouse CD16/32) in 198 μL of Cell staining Buffer (Biolegend).
36. **Carefully** remove the supernatant using a wide-bore 1 mL pipette tip first, and then completely remove the remaining supernatant with a 200 μL pipette tip. Be careful to not touch the tiny pellet.
37. Resuspend the pellet from each sample in 50 μL of blocking solution.
38. Incubate for 15′ at 4°C.
39. In the meantime, prepare the HTO-ADT antibody pool. Add 1 μL of each HTO Totalseq antibody to each sample (for cell hashing) and a titrated amount of each ADT antibody (for cell surface marker staining), according to manufacturer instructions.
  ***Note:*** we usually prepare a master mix solution for the ADT antibodies using saturating amounts of each antibody. For example, if you have 4 samples:
  a. Prepare 5 different 1.5 mL Eppendorf low bind tubes (4 tubes for each sample and 1 tube for the ADT antibody pool).
  b. Add 1 μL of a different HTO antibody to each of the 4 tubes.
  c. In the ADT antibody pool tube, add 2 μL of each ADT antibody and then add Cell Staining Buffer (Biolegend) up to a final volume of 196 μL. If you have > 100 ADT antibodies, no volume adjustment is necessary.
  d. Quickly spin down the ADT antibody pool at 8,000 × *g* for 15 s, and slowly pipette mix 5–6 times using a 200 μL pipette tip.
  e. Transfer 49 μL of the ADT antibody pool to each of the 4 tubes containing HTO antibodies and pipette mix.
  f. Quickly spin down the HTO-ADT antibody mixes at 8,000 × *g* for 15 s.
40. After 15′ of incubation in step 38, add 50 μL of each HTO-ADT antibody mix to each sample and slowly mix by pipetting up and down 5–6 times.
41. Incubate for 30′ at 4°C.
42. Add 300 μL of chilled Cell Staining Buffer (Biolegend) to each sample.
43. Spin down at 500 × *g* for 5′ at 20°C–25°C.
44. Carefully remove the supernatant without touching the cell pellet.
45. Repeat the washing step 42–44.
46. Resuspend each pellet in 150 μL of chilled DPBS.
47. Add 1,350 μL (final volume = 1.5 mL of 90% Methanol solution) of frozen Methanol to each sample drop-by-drop while rocking the tube in the meantime, to fix the cells.

***Note:*** we fix the cells at this stage, since the subsequent steps involving library generation and sequencing are performed outside the BSL3.

48. Store at −20°C for up to a week or at −80°C for long term storage.

### Rehydration of the fixed cells

**Timing: ∼40 min**

**CRITICAL:** we usually perform the following steps under a Biosafety hood. Clean all surfaces and pipettes with a product such as RNAse zap, to prevent degradation of RNA. Wear gloves at all times during the process. Maintain a clean and sterile work environment.

49. Decontaminate and pick up the methanol-frozen samples from the BSL-3 and equilibrate them on ice for 15’.
50. Prepare 2 mL (for 4 samples) of Rehydration buffer and keep on ice.
51. Spin down the methanol-frozen samples at 500 × *g*, 4°C for 10’.
52. Under the Biosafety hood, carefully remove and discard as much methanol as possible, without disturbing the cell pellet at the bottom of the tube.

***Note:*** carry over of even small traces of methanol into the 10× chip will lead to a failed run.

53. Quickly resuspend the pellet in 300 μL of Rehydration Buffer, by slowly pipetting up and down 10 times using a 200 μL pipette-tip.
54. Spin down at 500 × *g*, 10′ at 4°C.
55. Repeat wash steps 52–55.
56. Depending on the number of sorted cells, resuspend the pellet in 40 μL of Rehydration Buffer. We typically resuspend in 40 μL of Rehydration Buffer for every 60,000 sorted cells. Keep on ice.
57. Count the cells for each sample with a hemocytometer, withdrawing 10 μL volume from each sample.
58. Adjust the concentration of each sample to be between 700–1,200 cells/μL using Rehydration Buffer (this is the recommended input requirement for loading into the 10× chip).
  ***Note:*** at this point samples tagged with different HTOs can be combined in the desired proportions to multiplex into a single 10× run. If HTOs have *not* been used, then every sample needs to be run separately into a single 10× run.
  Alternative approach: HTO and ADT staining after methanol-fixation.
  a. Equilibrate methanol-fixed samples on ice for 15’.
  b. In the meantime, prepare HTO and ADT antibody pools as described above, using Cell Staining Buffer (Biolegend) containing 0.5 U/μL of RNase inhibitor.
  c. Spin down samples at 500 × *g*, 4°C for 10’.
  d. Remove as much methanol as possible without disturbing the cell pellet.
  e. Resuspend in 300 μL of Rehydration Buffer.
  f. Repeat wash step c-f.
  g. Spin down at 500 × *g*, 4°C for 10’.
  h. Remove as much rehydration buffer as possible.
  i. Resuspend the pellet from each sample in 50 μL of blocking solution (Cell staining Buffer containing 0.5 U/μL of RNase inhibitor + TruStain FCx plus).
  j. Incubate 15′ at 4°C.
  k. Add 50 μL of the HTO-ADT antibody mix to each sample and slowly pipette up and down for 5–6 times.
  l. Incubate for 30′ at 4°C.
  m. Add 300 μL of Cell Staining Buffer (Biolegend) containing 0.5 U/μL of RNase inhibitor.
  n. Spin down at 500 × *g* for 10′ at 4°C.
  o. Wash twice in 200 μL of Cell Staining Buffer containing 0.5 U/μL of RNase inhibitor, to remove all traces of non-bound antibodies.
  p. Resuspend in Cell Staining Buffer containing 0.5 U/μL of RNase inhibitor to a final concentration of 700–1,200 cells/μL, counting the cells for each sample on a hemocytometer.
  q. At this stage, samples tagged with different HTOs can be multiplexed in a single 10× run.

### Generation of CITE-seq libraries and sequencing

To generate CITE-seq libraries we follow the commercially available protocols from 10× (CG000206 Rev D) with the following modifications.

59. In step 1.1: we use the single-cell suspension generated in step 58 of the “rehydration of the fixed cells” section as the input for step 1.2b of the 10× protocol.

**CRITICAL:** first add the appropriate amount of Nuclease Free Water to the master mix and pipette mix 15 times, until the solution is homogeneous. *Do not add* nuclease free water to the single cell suspension.

**CRITICAL:** Pipette mix the single cell suspension 20 times before adding to the master mix, to breakup any potential clumps that could clog the chip and lead to a failed run.

60. In step 2.2a: we add 1 μL of ADT additive primer and 1 μL of HTO additive primer (if using both HTO and ADT) to the cDNA amplification mix. We also use the cDNA primers (included with the 10× kit) instead of the Feature cDNA Primers as highlighted in the 10× protocol, according to Biolegend recommendations.
61. For the library preparation for HTO and ADT antibodies we follow the Biolegend protocol without modifications.
62. For sequencing, we usually combine libraries (mRNA, HTO and ADT) from 2 samples on a single sequencing run on a NextSeq 2k. We typically allocate 80% of the total output reads to the mRNA libraries (split accordingly to the number of cells loaded in the 10× chip for each sample), 10% for the HTO libraries and 10% for the ADT libraries. We perform paired-end sequencing with the following parameters: 28 bp Read 1, 8 bp Index I7, 56 bp Read 2.

## Expected outcomes

Upon execution of the above protocol, the user will generate 3 different libraries (mRNA, HTO, ADT) each containing different indexes that will be pooled and sequenced in a single sequencing run.

We always pool the libraries such that at least 85% of all reads belong to the mRNA library. If using ≤ 20 ADT/HTO markers, we pool libraries using the following proportions: 90% mRNA, 5% ADT, 5% HTO; if using > 20 ADT/HTO markers we pool using the following proportions: 85% mRNA, 10% ADT, 5% HTO.

We allocate an entire Nextseq 500 flow cell (∼400 M reads) for each 10× sample containing 10,000 cells. When pooling multiple 10× samples in a single sequencing run we either use an entire NextSeq 2k or Novaseq S4 lane, depending on the amount of samples and desired reads output.

Before sequencing, quality of the generated libraries should be assessed by Fragment Analyzer. Users should expect to see a distribution of fragments for the mRNA libraries (300–600 bp as described in the 10× commercially available protocol CG000206 Rev D) with only minor peaks under 200 bp. For the HTO and ADT libraries users should expect to observe a single peak centered at ∼210 bp.

The Mtb-infected lung samples contain high proportions of different macrophage populations: we typically recover an average of 800–1,200 genes/cell using the methanol-fixation protocol illustrated above.

After data analysis, users should be able to recover and clearly separate different populations of alveolar and interstitial macrophages.

## Limitations

This protocol has been developed to use with the 10× scRNA-seq 3′ Gene Expression kits. We have *not tested* whether our protocol is compatible with the 5′ scRNA-seq immune repertoire kits from 10×. Furthermore, our protocol is not compatible with scATAC-seq because of the methanol fixation step, which will degrade the chromatin organization structure of the nuclei.

Our protocol involves fluorescent activated cell sorting of live material to isolate infected from bystander cells, which may not be an available option in all laboratories. Finally, our protocol requires the use of fluorescent reporter strains to sort infected cells based on the fitness of the bacterial pathogen, which while relatively available for *M. tuberculosis*, may not be available for other infectious agents.

## Troubleshooting

### Problem 1

Low number of cells recovered for input into the 10× chip in step 58 of the “rehydration of the fixed cells” section.

### Potential solution

• Increase the number of sorted cells. We found 60,000 sorted cells to be a reasonable compromise with respect to the duration of sorting time and number of cells that are recovered at the end of the protocol for input into the 10× chip.
• Be careful not to disturb the pellet during the washing steps of the “Methanol fixation” and “rehydration of the fixed cells” parts of the protocol. The pellet generated from 60,000 cells will be visible at the bottom of the 1.5 mL Eppendorf tubes. Disturbing the pellet during the cell wash steps will lead to decreased cell recovery.

### Problem 2

Failed 10× run resulting in a wetting failure or reagent clog.

### Potential solution

• Make sure to resuspend the single-cell suspension pipetting up and down at least 20 times before adding to the Master mix. We also suggest resuspending the single cell suspension plus master mix at least 15 times before loading in the 10× chip.
• Make sure no bubbles are present into Row 1 and Row 2 of the 10× chip after loading of the reagents, before inserting the chip into the controller.
• Make sure to not touch the bottom of the 10× chip, to avoid creating static electricity which can result in a failed run.

### Problem 3

High amount of background RNA among the samples, which will lead to low numbers of genes/cell detected, high number of empty “cells” and <30% reads matching the reference sequence.

### Potential solution

• Make sure that your infection model does not induce extensive necrosis among your samples. We tried sorting samples from RAG1 KO mice, which 3 weeks post Mtb infection had high amounts of necrotic tissue, and we found that elevated levels of background RNA were present in the samples, resulting in unusable 10× runs.
• Try to avoid tissues containing large numbers of cells with low RNA content, such as neutrophils, which will lyse during the execution of this protocol, resulting in release of background RNA in the samples.
• Thoroughly clean the sorter before use and let a solution of RNAse zap run through the sample line for 5′, to avoid carryover of background RNA and RNAse (which will degrade your samples) from previous usage.

### Problem 4

Low/high yields of the mRNA libraries < 1 ng or > 200 ng.

### Potential solution

• Adjust the PCR cycle numbers in the “cDNA amplification” step 2.2 and “Sample Index PCR” step 3.5 of the 10× manual. Make sure to increase/decrease the amount of PCR cycles depending on the type of immune cells that are prevalent in your sample (high/intermediate/low RNA content).
• Make sure to count and to load the correct number of cells in the master mix step 1.2 of the 10× manual. For example, if we target to recover 10,000 cells, but we only load 4,000 in the chip and we setup the number of PCR cycles in the cDNA amplification step 2.2 such as we loaded 10,000 cells, the resulting yields for the mRNA libraries will be low.
• Make sure that > 80% of the cells in your sorted samples are viable at the time of methanol fixation.

### Problem 5

Low complexity of the mRNA libraries.

### Potential solution

• Adjust the PCR cycle numbers in the “cDNA amplification” step 2.2 of the 10× manual to avoid overamplification of specific cDNA sequences.

### Problem 6

Presence of a significant peak < 100 bp in the HTO, ADT and mRNA libraries.

### Potential solution

• This is usually due to carryover of adapter primers in the sequencing reaction. Perform a 1× SPRI beads cleanup on the mRNA library and/or a 1.2× SPRI beads cleanup on the ADT and HTO libraries, to remove any peak < 100 bp.

## Resource availability

### Lead contact

Further information and requests for resources and reagents should be directed to and will be fulfilled by the lead contact, David G. Russell (dgr8@cornell.edu).

### Materials availability

This study did not generate new unique reagents.

## Data Availability

The raw datasets needed to repeat the analysis published in J Exp Med are available on GEO: https://www.ncbi.nlm.nih.gov/geo/query/acc.cgi?acc=GSE167232.
